# Integrating Pain Prehabilitation into Surgical Pathways: Current Modalities, Outcomes, and Research Gaps

**DOI:** 10.1007/s11916-025-01464-2

**Published:** 2026-02-09

**Authors:** Eric A. Sosa, Anabel Henick, Dhanesh D. Binda, Crystal Joseph, Stanley Kim, Dave Mathew, Singh Nair, Jinu Kim, David C. Adams, Karina Gritsenko, Alan D. Kaye, Ugur Yener, Hatice Begum Ciftci, Sayed E. Wahezi, Naum Shaparin

**Affiliations:** 1 Department of Anesthesiology, Montefiore Medical Center, 111 E. 210Th Street, Bronx, NY 10467 USA; 2 Department of Anesthesia, Beth Israel Deaconess Medical Center, Boston, MA USA; 3 Department of Emergency Medicine, Icahn School of Medicine at Mount Sinai, New York, NY USA; 4 Rutgers Health/Community Medical Center, Toms River, NJ USA; 5 Department of Anesthesia, Indiana University School of Medicine, Indianapolis, IN USA; 6 Department of Anesthesia, Louisiana State University School of Medicine, Shreveport, LA USA; 7 Department of Rehabilitation Medicine, Montefiore Medical Center, Bronx, NY USA

**Keywords:** Prehabilitation, Pain management, Perioperative pain, Anesthesiology, Surgery, Enhanced recovery, Perioperative medicine

## Abstract

**Purpose of Review:**

Prehabilitation constitutes a multidisciplinary strategy aimed at improving physiological and psychological readiness prior to surgery. Within pain medicine, prehabilitation provides an opportunity to address modifiable pain-related and functional risk factors prior to major procedures. This narrative review synthesizes contemporary evidence on prehabilitation modalities, their impact on surgical and pain-relevant outcomes, and persistent knowledge gaps limiting integration into perioperative pain pathways.

**Recent Findings:**

A targeted PubMed search was performed on June 25, 2025. Supplementary exploratory searches in Web of Science and Scopus did not identify unique eligible studies beyond those already captured in PubMed. After excluding non-original reports and studies lacking outcome data, 153 clinical studies were included. The most frequently represented surgical specialties were general surgery (43%), orthopedic surgery (21%), and cardiothoracic surgery (18%). Single-modality prehabilitation was reported in 43% of studies, whereas multimodal approaches varied by specialty, occurring in 36% of general surgery studies and 13% of cardiothoracic surgery studies. Five principal prehabilitation modalities were identified: exercise, nutrition, psychological intervention, substance cessation, and medical optimization. Exercise-based interventions were the most common, incorporated in 84.7% of studies, followed by nutritional interventions in 29.5%. Overall, 82% reported improvements in at least one postoperative or functional outcome, although pain-specific endpoints were inconsistently reported.

**Summary:**

Prehabilitation appears beneficial across surgical specialties; however, pain outcomes remain underreported, and multimodal programs remain limited. Future work should standardize pain endpoints and evaluate multimodal interventions to guide integration of prehabilitation into perioperative pain management pathways.

**Supplementary Information:**

The online version contains supplementary material available at 10.1007/s11916-025-01464-2.

## Introduction

Population projections indicate that the number of individuals aged 65 years and older will double within the next decade [1]. This demographic shift, coupled with age-related physiological decline and the increasing prevalence of frailty, is expected to place substantial strain on perioperative services and heighten the burden of postoperative complications, including poorly controlled pain, prolonged opioid use, and delayed functional recovery [2, 3]. These trends underscore the growing importance of prehabilitation strategies aimed not only at improving surgical resilience but also at optimizing perioperative pain trajectories in vulnerable populations.

Frailty, a multidimensional syndrome characterized by diminished physiologic reserve and impaired capacity to respond to surgical stress, is a major determinant of postoperative morbidity and mortality and is increasingly recognized as a contributor to adverse pain outcomes, including heightened pain sensitivity, impaired pain coping, and prolonged recovery [4]. In older and deconditioned patients, the physiologic stress response to surgery is strongly associated with increased postoperative complications and mortality, as well as greater pain intensity and analgesic requirements [5–7]. These risks are amplified by common comorbidities, such as cardiovascular disease, chronic pulmonary disease, diabetes, chronic kidney disease, malnutrition, and cognitive impairment, all of which may influence pain perception, analgesic response, and recovery [8]. Although advances in perioperative medicine have improved outcomes in this population, postoperative pain remains a persistent and clinically consequential challenge shaped by physical, psychological, and social determinants of health. Traditionally, postoperative rehabilitation has been emphasized as the primary means of recovery; however, its ability to meaningfully alter pain-related outcomes has been increasingly questioned [9–15].

The concept of “prehabilitation” was first introduced in 1946 by the British military to describe interventions designed to correct physical deficits prior to fitness evaluations [16], with renewed scientific interest emerging in the 1980 s within sports medicine [17]. In contemporary perioperative care, prehabilitation encompasses structured interventions delivered before surgery to optimize physical, nutritional, and psychological readiness. Within this framework, pain-focused prehabilitation refers to strategies intended to modify pain-related risk factors prior to surgery, with the goal of improving postoperative pain control, reducing opioid exposure, and mitigating the development of chronic postsurgical pain [18]. In this review, “pain prehabilitation” does not denote a distinct class of interventions, but rather an outcome-focused framework through which established prehabilitation modalities are evaluated for their effects on pain-related outcomes when such outcomes are reported. While the term “pain prehabilitation” has appeared in the literature, pain is rarely evaluated as a primary endpoint; instead, pain-related constructs—such as baseline discomfort, mobility limitation, catastrophizing, anxiety, and functional restriction—are often embedded within broader measures of surgical readiness or recovery.

Common prehabilitation interventions include aerobic and resistance exercise programs, nutritional optimization, respiratory training, and psychological support, such as stress reduction and counseling. Notably, several of these modalities—particularly exercise and psychological interventions—have demonstrated independent analgesic and pain-modulating effects in non-surgical populations, suggesting a plausible mechanistic role in shaping perioperative pain outcomes. Poor preoperative physical conditioning and psychological distress have consistently been linked to worse postoperative pain and prolonged recovery, positioning prehabilitation as a potentially modifiable upstream target [19–21].

To date, prehabilitation research has been concentrated largely within colorectal surgery [22–25], with expanding interest across urologic, gynecologic, orthopedic, and cardiothoracic procedures [26, 27]. Prior studies suggest that prehabilitation can improve physical function, reduce postoperative complications, and shorten hospital length of stay [28, 29]. However, whether these benefits translate into meaningful improvements in perioperative pain, such as reduced pain intensity, opioid consumption, or chronic pain risk, remains unclear due to inconsistent outcome reporting and heterogeneity in study design.

Despite increasing recognition of its potential value, pain-focused prehabilitation has not been widely adopted as standard practice, and pain-specific outcomes remain inconsistently reported across the literature [30]. As a result, optimizing pain prior to surgery remains an underexplored but clinically important target. This narrative review, therefore, aims to critically examine the existing prehabilitation literature through a pain-focused lens, emphasizing how current interventions may influence perioperative pain outcomes, identifying gaps in pain-specific reporting, and highlighting opportunities for future research. By reframing prehabilitation as both a strategy for enhancing surgical readiness and a means of optimizing pain trajectories, we seek to inform more comprehensive, patient-centered approaches to perioperative care.

## Methods

### Search Strategy

A structured search was conducted on June 25, 2025, using PubMed, Scopus, and Web of Science to identify studies examining prehabilitation interventions in surgical populations. Search terms included “prehabilitation AND anesthesia,” “prehabilitation AND surgery,” “prehabilitation AND preoperative medicine,” and “prehabilitation.” No date restrictions were applied. Records retrieved from multiple databases were deduplicated prior to screening. The search strategy was intentionally broad to capture studies explicitly describing preoperative interventions intended to enhance surgical readiness.

### Study Selection

Two investigators (EAS and AH) independently screened articles identified through the specified search strategy to determine eligibility. Eligible studies included randomized and non-randomized clinical trials, as well as retrospective analyses, evaluating preoperative interventions explicitly described as prehabilitation. Studies were excluded at full-text review if the intervention was not explicitly described as prehabilitation, consisted solely of perioperative or postoperative care, or involved preoperative interventions without conceptual framing as prehabilitation. Studies incorporating Enhanced Recovery After Surgery (ERAS) pathways were included only when a clearly defined preoperative prehabilitation component was described and could be distinguished from perioperative or postoperative ERAS elements; studies examining ERAS protocols without a separable preoperative intervention were excluded. Feasibility studies and studies evaluating prehabilitation combined with postoperative rehabilitation were included when preoperative effects could be reasonably isolated. Additional exclusion criteria encompassed systematic reviews, meta-analyses, general reviews, protocol descriptions, case reports, opinion pieces, and letters to the editor. Pain was not used as an inclusion criterion due to inconsistent reporting across the literature; however, pain-related outcomes were extracted when available. Any intervention delivered prior to surgery and explicitly described as prehabilitation was eligible regardless of duration, modality, or timing relative to the scheduled procedure.

### Data Extraction

For each included study, the following data were extracted: PubMed ID, article title, year of publication, surgical specialty, type of surgery, study design, prehabilitation modality or modalities, inclusion of cancer patients, receipt of neoadjuvant chemotherapy, sample size, mean age, gender distribution, duration of prehabilitation, involvement of ERAS protocols, number lost to follow-up, primary and secondary outcomes, data collection methods, and study conclusions. Pain-related outcomes—including postoperative pain intensity, analgesic or opioid consumption, pain-related functional limitation, and chronic postsurgical pain—were extracted when reported; pain was not used as an inclusion criterion due to inconsistent reporting across studies, but its presence, timing (short-term < 3 months vs long-term > 3 months), and direction of effect were recorded when available. Cardiothoracic surgeries were subcategorized into two groups: Group 1 A included true cardiac procedures (e.g., coronary artery bypass grafting and valve replacements), while Group 1B encompassed oncologic procedures (e.g., esophagectomies and pulmonary resections). Cancer or neoadjuvant chemotherapy status was recorded only when explicitly stated. Study conclusions were classified as “positive,” “negative,” or “equivocal” based on statistical significance and authors’ interpretations. No additional statistical analyses, pooled estimates, or weighting by study design were performed; results were summarized descriptively to characterize patterns across the literature rather than to infer comparative efficacy. All data are reported as counts and proportions, and analysis was conducted using Microsoft Excel Version 16.46. For interpretative clarity, studies classified as “negative” or “equivocal” were grouped together. The presence of ERAS pathways was recorded as a co-intervention when applicable; however, outcomes were not attributed to ERAS components, and ERAS was not classified as a prehabilitation modality.

### Categories of Prehabilitation

Prehabilitation regimens were classified into five primary categories: exercise, nutritional supplementation, psychological support, substance cessation, and medical optimization. The exercise category encompassed all forms of physical activity or training, including physical therapy, aerobic exercise, resistance or strength training, stretching, and walking; for urological studies, continence and pelvic floor training were also included. Nutritional prehabilitation involved interventions such as modified dietary regimens, nutrition education, or consultations with a registered dietitian. Psychological support interventions included therapy sessions, emotional support groups, motivational interviewing, and anxiety management strategies, and were distinguished by delivery format (in-person versus virtual) when explicitly reported, as this modality was most consistently differentiated by delivery method across studies. Delivery format for other modalities was not uniformly reported and therefore was not systematically classified. Substance cessation encompassed counseling aimed at discontinuing alcohol, tobacco, or opioid use. Medical optimization referred to interventions such as preoperative antibiotic regimens, improvement of glycemic control (e.g., reduction in glycosylated hemoglobin), anemia correction, and frailty management; neoadjuvant chemotherapy was not considered a form of medical optimization but was recorded when reported.

The number of prehabilitation modalities used in each study was determined by the number of distinct categories represented in the intervention. Regimens incorporating two or more categories—such as exercise combined with nutritional support or psychological counseling—were classified as multimodal, reflecting integrated approaches designed to address multiple physiological and psychosocial domains to enhance surgical readiness and recovery.

## Results

### Types of Studies Analyzed

The preliminary screening process identified 177 full-text articles for evaluation. Following full-text review, 24 articles were excluded due to irrelevance or lack of reported outcomes, yielding a final cohort of 153 studies for comprehensive analysis (Supplementary Table 1). Of these, 45% were randomized controlled trials, 37% were prospective non-randomized cohort studies, 10% were retrospective reviews, and 5% represented other study designs. Although most studies evaluated perioperative outcomes broadly, pain-specific endpoints were variably reported and infrequently designated as primary outcomes.

### Publication Timeline

**Fig. 1:**
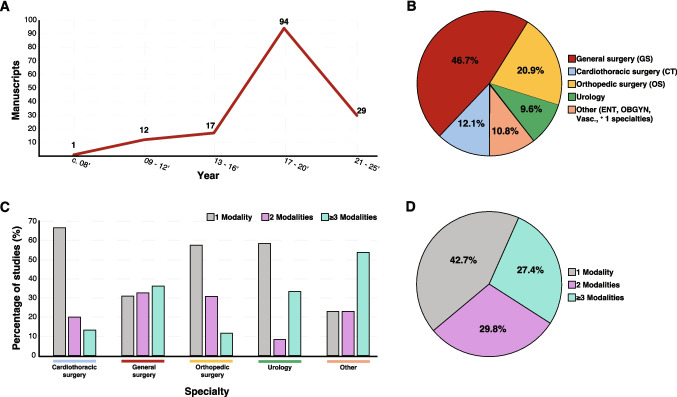
Temporal trends, specialty distribution, and modality composition of prehabilitation manuscripts. **A** Prehabilitation manuscripts between 2008 and 2025 (x-axis) indicate an increase in prehabilitation modality usage (y-axis). The x-axis representing the year of publication was divided into three-year increments. In 2008, there was 1 publication, increasing to 12 between 2009–2012, 17 between 2013–2016, and a surge between 2017–2020 with 94 manuscripts applying prehabilitation modalities. **B** Pie chart highlighting the specialty composition of the 153 studies analyzed. General surgery (red) comprised the greatest proportion at 46.7%, followed by orthopedic surgery at 20.9% (yellow), cardiothoracic surgery at 10.8% (blue), urology at 9.6% (green), and the Other category (ENT, obstetrics/gynecology, vascular, and multispecialty) at 12.1% (tan). The “Other” category included studies spanning more than one specialty. **C** Representation of the number of prehabilitation modalities employed in the analyzed studies by specialty. The use of one, two, or three or more prehabilitation modalities is represented by grey, purple, and blue bars, respectively. The percentage of studies employing these modalities is shown on the y-axis. **D** Pie chart visual representation of the number of modalities used in the analyzed studies in aggregate. 42.7% of studies used one modality, 29.8% used two, and 27.4% utilized three or more modalities

Among the 153 included studies, 124 (81%) were published between 2017 and 2020, and 29 (19%) between 2021 and 2025. The earliest clinical trial meeting the inclusion criteria was published in 2008. Publication volume increased substantially over time, with 12 studies published between 2009 and 2012, 17 between 2013 and 2016, and 123 between 2017 and 2025 (Fig. 1A). This growth reflects expanding academic interest in prehabilitation, though the inclusion of standardized pain-related outcomes and implementation metrics has not increased proportionally over time.

### Demographic Characteristics

Across all studies, the mean patient age was 62.4 years (SD = 11.6; range 23.0–80.6), representing a population at heightened risk for postoperative pain, prolonged opioid exposure, and chronic postsurgical pain. Orthopedic surgery studies included the youngest patients (mean = 60.3 years; SD = 14.7), while urology studies included the oldest (mean = 65.8 years; SD = 4.7). Cardiothoracic and general surgery studies reported mean ages of 64.0 years (SD = 5.8) and 61.8 years (SD = 12.8), respectively.

The mean sample size was 130.9 patients (SD = 230.8), with wide variability driven by differences in study design. Sample sizes ranged from six patients in small interventional trials to 2,187 in retrospective analyses. Orthopedic surgery studies demonstrated the greatest variability in sample size (SD = 351.3), while cardiothoracic surgery studies showed the least (SD = 85.7). Gender distribution favored male participants overall (male-to-female ratio 1.4:1.0), with cardiothoracic surgery exhibiting the highest male predominance (2.1:1.0) and orthopedic surgery the lowest (0.8:1.0).

### Adherence, Follow-up, and Engagement Reporting

Loss to follow-up was reported in 96 of the 153 studies, with a mean follow-up completion rate of 85.2% (SD = 14.7%). Cardiothoracic surgery studies demonstrated the highest follow-up completion rates (mean = 89.1%; SD = 11.2%), whereas studies spanning multiple surgical specialties reported the lowest (mean = 81.0%; SD = 21.8%). While follow-up completion serves as a pragmatic marker of retention, more granular measures of adherence to prehabilitation interventions—such as session attendance, completion of prescribed exercise regimens, or engagement with multimodal components—were inconsistently reported. When included, adherence and engagement metrics were variably defined, limiting cross-study comparison and quantitative synthesis. Nonetheless, reporting of follow-up and retention outcomes suggests that prehabilitation programs are feasible in selected populations, while highlighting the need for more standardized implementation metrics.

The mean duration of prehabilitation programs across studies was 5.8 weeks (SD = 4.7), a timeframe relevant to potential modulation of baseline pain, physical conditioning, and psychological readiness prior to surgery.

### Oncologic Context

More than half of the included studies (56.3%) focused on patients undergoing surgery for malignancy, a population at increased risk for complex pain trajectories due to tumor burden, prior treatments, and psychological stressors. However, only 23.5% of studies explicitly included patients receiving neoadjuvant chemotherapy, which may further influence pain sensitivity, analgesic requirements, and engagement with prehabilitation programs. Orthopedic surgery studies did not include oncologic populations, whereas urology studies demonstrated the highest proportion of cancer-focused prehabilitation (80.0%), largely in prostate and bladder cancer resections.

### Surgical Specialties

The most frequently represented surgical specialties were general surgery (46.7%), orthopedic surgery (20.9%), cardiothoracic surgery (12.1%), and urology (9.6%) (Fig. 1B; Supplementary Table 1). Studies from specialties with limited representation—including otolaryngology, vascular surgery, obstetrics and gynecology, and multidisciplinary cohorts (10.8%)—were grouped as “Other Studies.”

Within general surgery, colorectal procedures predominated (65.5%), representing a population commonly studied for functional recovery and postoperative pain but with heterogeneous pain outcome reporting. In orthopedic surgery, joint replacement (50.0%) and spine surgery (30.7%)—procedures with well-recognized pain burdens—were most frequently examined. Cardiothoracic studies focused primarily on pulmonary (33.3%) and esophageal resections (26.6%), while urologic studies centered on prostatectomy (58.3%) and cystectomy (41.6%). Less frequently studied procedures included organ transplantation, coronary artery bypass grafting, hepatopancreaticobiliary surgery, and hernia repair.

### Patterns of Prehabilitation Modality Use Across Surgical Specialties

Studies were analyzed according to the number of prehabilitation modalities employed across surgical specialties (Fig. 1C). In cardiothoracic surgery, 67% of studies used a single modality, while 20% and 15% employed two or ≥ 3 modalities, respectively. General surgery demonstrated a more even distribution, with 31% using a single modality, 33% using two, and 37% employing ≥ 3 modalities—suggesting greater uptake of multimodal approaches that may address both physical conditioning and pain-related risk factors.

Orthopedic and urologic studies predominantly utilized single-modality interventions (approximately 60% each), despite these populations being at high risk for postoperative pain. In contrast, the pooled “other” specialties demonstrated the greatest use of ≥ 3 modalities (54%), exceeding single- and dual-modality approaches (22% each). Across the full cohort, 42.7% of studies employed a single modality, 27.4% used two modalities, and 29.8% utilized ≥ 3 modalities (Fig. 1D). While multimodal prehabilitation is conceptually aligned with multimodal pain management, its adoption remains inconsistent across specialties.

### Types and Prevalence of Prehabilitation Modalities

**Fig. 2:**
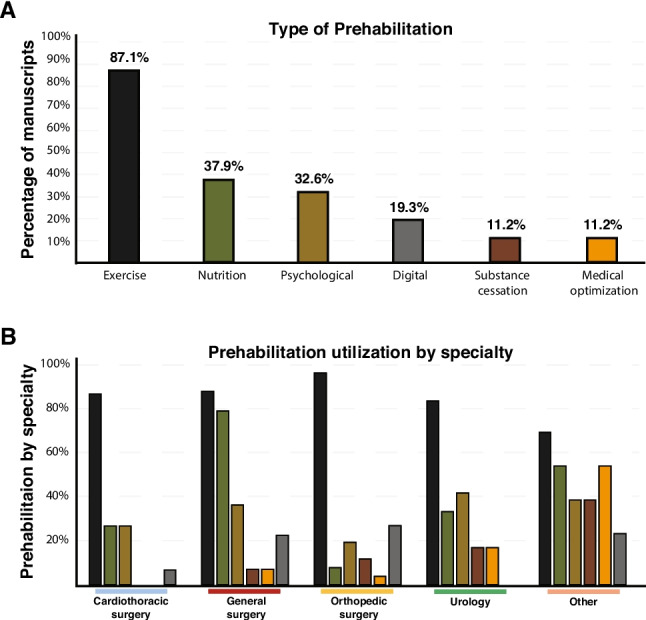
Distribution of prehabilitation modalities and utilization by surgical specialty. **A** Percentage of manuscripts that reported each prehabilitation modality (y-axis); bars were color-coded according to the following scheme: exercise (black, 87.1%), nutrition (green, 37.9%), psychological (light brown, 32.6%), digital (grey, 19.3%), substance cessation (dark brown, 11.2%), and medical optimization (orange, 11.2%); modalities were ordered by frequency, with exercise the most utilized. **B** Prehabilitation utilization subdivided by surgical specialty (x-axis: cardiothoracic surgery, general surgery, orthopedic surgery, urology, other) with the percentage of studies on the y-axis; bars were colored by modality according to the scheme in A and applied consistently across the panel

Five categories of prehabilitation were identified: exercise, nutritional support, psychological interventions, substance cessation, and medical optimization. Exercise was the most employed modality, present in 87.1% of studies, followed by nutritional interventions (37.9%) and psychological support (32.6%)—both of which have established associations with pain perception, coping, and recovery. Medical optimization and substance cessation were each included in 11.2% of studies (Fig. 2A).

Overall, 59% of studies utilized a single modality (Supplementary Table 1). Multimodal prehabilitation—defined as the integration of two or more intervention categories—was employed in 20% of studies, with notable variation by specialty. General surgery had the highest use of multimodal regimens (27%), whereas cardiothoracic surgery had the lowest (10%) (Fig. 2B). The mean number of modalities per study was 1.98, ranging from 2.12 in general surgery to 1.46 in cardiothoracic surgery. Despite frequent inclusion of exercise and psychological components, few studies explicitly evaluated their effects on pain-related outcomes.

### Economic Outcomes and Resource Use

Economic outcomes were infrequently reported across the included studies. When present, analyses varied substantially in scope and methodology, including measures of hospital length of stay, readmissions, direct costs, or cost-effectiveness. However, inconsistent reporting and heterogeneity in analytic approaches precluded quantitative synthesis of economic impact. These findings indicate that, despite increasing interest in prehabilitation, evidence regarding its financial implications and resource utilization remains limited and insufficiently standardized.

### Proportion of Studies Reporting Improvement in Clinical and Pain-Related Outcomes

**Fig. 3:**
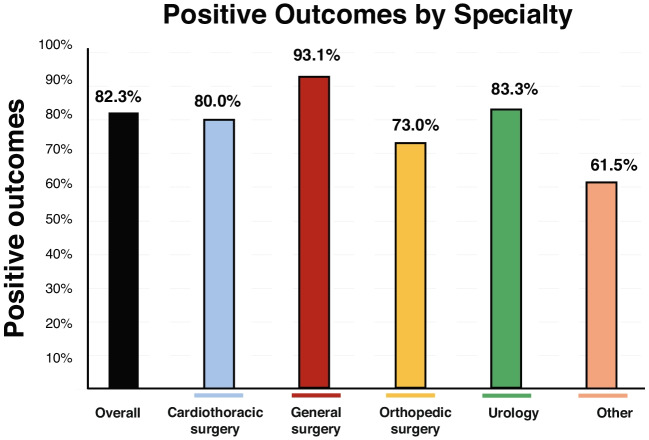
Positive outcomes associated with prehabilitation by surgical specialty. **A** Proportion of included studies reporting positive outcomes (y-axis, %) presented overall and stratified by specialty (x-axis). Positive outcomes were observed in 82.3% overall; by specialty: cardiothoracic surgery, 80.0%; general surgery, 93.1%; orthopedic surgery, 73.0%; urology, 83.3%; and other, 61.5%. Percentages were calculated from the included dataset (*n* = 153); “other” comprised ENT, obstetrics/gynecology, vascular, and multispecialty studies

Across all included studies, 82% reported statistically significant improvement in at least one predefined clinical outcome, most commonly functional capacity, postoperative complication rates, or hospital length of stay (Fig. 3). Pain-related outcomes, including postoperative pain intensity, analgesic consumption, or pain-associated functional limitations, were reported less consistently and were infrequently designated as primary endpoints. When assessed, pain-related improvements generally occurred alongside gains in functional or recovery-related measures rather than as isolated outcomes. The proportion of studies reporting improvement in at least one clinical outcome varied by surgical specialty, with general surgery demonstrating the highest rate (93.1%), followed by urology (83.3%), cardiothoracic surgery (80.0%), and orthopedic surgery (73.0%) (Supplementary Table 1). These findings reflect outcome-specific improvements rather than uniform benefit across all measured domains.

### Short- and Long-Term Pain-Related Outcomes

To further characterize the temporal effects of prehabilitation, outcomes were stratified by follow-up duration. Short-term outcomes were defined as those assessed within 3 months postoperatively, while long-term outcomes were defined as those assessed beyond 3 months.

Among the 153 included studies, the majority (125/153; 82.3%) reported a statistically significant improvement in short-term pain-related outcomes following prehabilitation. Two studies (2/153; 1.3%) demonstrated neutral effects, while 21 studies (21/153; 13.7%) reported no significant short-term improvement in pain outcomes compared with control groups receiving no prehabilitation.

In contrast, long-term pain-related outcomes were reported far less frequently. Only 22 of 153 studies (14.4%) evaluated pain beyond 3 months postoperatively. Among these, 12 studies demonstrated significant long-term improvement in pain-related outcomes, while the remaining studies reported no significant differences compared with controls. The limited number of studies assessing long-term pain outcomes precluded formal synthesis and underscores a substantial gap in the existing literature.

## Discussion

Since 2008, research on pain prehabilitation has expanded rapidly, with most studies in our review published between 2017 and 2025, underscoring prehabilitation as a relatively recent focus in perioperative care. The present study demonstrates that, despite frequent inclusion of prehabilitation across surgical specialties, pain-related outcomes are inconsistently reported, rarely designated as primary endpoints, and predominantly assessed in the short term, limiting conclusions regarding the durability and specificity of pain-focused prehabilitation effects. Together, these findings suggest that pain-focused prehabilitation remains incompletely characterized within the existing literature and highlight the need for more consistent, pain-centered outcome assessment. Since this narrative review synthesizes evidence across heterogeneous study designs, results should be interpreted as descriptive trends rather than comparative estimates of efficacy.

Analysis of demographic data revealed a predominance of male participants across studies, with an overall male-to-female ratio of 1.4:1.0. This contrasts with findings from a previously published review of seven studies, in which five demonstrated gender parity, and two showed a female predominance [31]. The most pronounced gender disparity was observed in cardiothoracic surgery studies, where the male-to-female ratio reached 2.1:1.0. This likely reflects the higher incidence of lung cancer in men, as pulmonary resections—commonly indicated for lung malignancies—represented the most frequently studied procedure within this specialty [32]. The mean age of participants was in the elderly range, aligning with the increased surgical risk, frailty, and comorbidity burden typically observed in older populations [32–34]. Given that most studies reporting positive outcomes involved elderly and predominantly male patients, these populations may stand to derive the greatest benefit from prehabilitation interventions.

### Adherence and Patient Engagement

Across all studies analyzed, the average rate of adherence at follow-up was 85.2%. Notably, cardiothoracic surgery studies reported the highest adherence rates. This is particularly compelling given that cardiac surgery patients typically present with multiple comorbidities, which are known to contribute to higher perioperative risk and potential loss to follow-up [25]. Several factors may explain this elevated adherence. Patients undergoing cardiothoracic procedures, such as coronary artery bypass grafting (CABG) or valve replacement, may have a heightened awareness of the risks associated with their condition and the complexity of postoperative recovery. This awareness, possibly driven by fear or a clearer understanding of the severity of their disease, may enhance motivation to engage with prehabilitation and follow-up protocols. Additionally, evidence suggests that prehabilitation in cardiac patients yields substantial benefits. In a recent observational study evaluating prehabilitation prior to CABG, participants demonstrated significant improvements in cardiorespiratory fitness, body mass index, and psychological outcomes, such as symptoms of depression and anxiety, alongside increased participation in postoperative cardiac rehabilitation programs [26]. These findings indicate that prehabilitation not only improves measurable health outcomes but may also foster sustained patient engagement in long-term health optimization strategies.

### Prevalence of Pain Prehabilitation Across Surgical Specialties

From our analysis, the primary surgical specialties utilizing prehabilitation, in order of frequency, were general surgery, orthopedics, cardiothoracic surgery, and urology. Previous systematic reviews have demonstrated similar distributions [35–37]. Across the literature, five core prehabilitation modalities were identified: exercise, nutritional planning, psychological intervention, substance cessation, and medical optimization [38, 39]. Despite this breadth, most studies implemented only a single modality, with exercise being the most frequently employed. The mean number of modalities per study was 1.7, which likely reflects the disproportionate impact of the relatively small number of studies that incorporated several interventions, thereby raising the average above one. Given the evidence that multimodal prehabilitation can reduce morbidity and mortality, this represents a critical opportunity for improvement [40, 41]. Additional research is warranted to assess the efficacy of multimodal interventions. By incrementally integrating multiple components, pain prehabilitation could be delivered in a comprehensive, single-visit framework, yielding a more holistic, efficient, and clinically effective approach.

### Targeted Subspecialty Approaches

In some subspecialties, prehabilitation modalities were highly targeted. For example, several urological studies employed pelvic floor muscle training to mitigate post-procedural urinary incontinence, while select otolaryngological studies administered gentamicin to accelerate vestibular function recovery [42–45]. These examples illustrate how refined, indication-specific prehabilitation strategies may prevent common complications in defined patient subsets. Future research aimed at identifying prevalent complications and matching them with targeted interventions could inform best practices for subspecialty-specific prehabilitation.

### Clinical Outcomes and Knowledge Gaps

The literature predominantly demonstrates positive postoperative outcomes associated with prehabilitation. Improvements in functional capacity and quality of life were consistently reported in general surgery, cardiothoracic surgery, and urology [28, 30]. Despite these encouraging results, important knowledge gaps remain. As a relatively novel area of investigation, additional studies are needed to further delineate the role of prehabilitation within the framework of ERAS pathways, which have achieved widespread adoption across most surgical specialties [43, 46].

### Underutilized Specialties and Disparities

Several surgical subspecialties, including neurosurgery, otolaryngology, and vascular surgery, rarely employed prehabilitation. The limited uptake in these fields represents another opportunity for exploration. The reasons underlying this underutilization remain unclear but may include patient-level barriers or the urgency associated with operative intervention. Additionally, demographic disparities were evident, with younger patients and female patients underrepresented in published cohorts. Addressing these gaps in future research will be essential to ensure prehabilitation protocols are representative of the broader surgical population.

### Risk Stratification and Long-Term Outcomes

Incorporating temporal outcome assessment into prehabilitation research represents an important area for future advancement [44]. While most studies in this review focused on short-term outcomes (< 3 months), over 80% reported significant short-term improvements in pain-related outcomes following prehabilitation. In contrast, long-term pain outcomes (> 3 months) were infrequently assessed, with fewer than 15% of studies reporting extended follow-up. Among these, approximately half demonstrated sustained pain-related benefit, while the remainder showed no significant differences compared with controls. This imbalance limits conclusions regarding the durability of pain prehabilitation effects and highlights a critical gap in the current evidence base. Given the clinical relevance of chronic postsurgical pain, future studies should prioritize standardized long-term pain outcomes to better define the sustained role of prehabilitation in perioperative care. These findings underscore the need to consider both procedural risk and follow-up duration when evaluating the clinical impact of prehabilitation.

Incorporating surgical risk stratification into prehabilitation studies represents another potential area of advancement [44]. Many studies combined heterogeneous surgical practices, complicating efforts to determine whether procedural risk influenced prehabilitation outcomes. Stratification by procedural invasiveness could better clarify the relationship between prehabilitation and clinical endpoints. Furthermore, while most studies focused on short-term outcomes (< 3 months), the long-term durability of pain prehabilitation benefits remains poorly characterized. Investigation into long-term outcomes will be critical to defining its sustained clinical value.

### Economic Implications

Evidence regarding the financial implications of pain prehabilitation is limited and inconsistent. Few studies have directly assessed its cost-effectiveness, as interventions are often initiated exclusively within research contexts. Since prehabilitation is not yet a standard of care, it is not routinely prescribed, and therefore, limited guidance exists regarding its cost–benefit balance. While some studies have reported cost savings, others have demonstrated neutral or unfavorable findings [47–49]. For example, a 2019 cost-analysis reported no significant savings at 30 days among patients enrolled in a prehabilitation program [49]. Further research is required to elucidate the economic impact of prehabilitation, particularly in real-world settings.

### Digital Delivery Models

Implementation of prehabilitation also entails considerable resource demands and patient time commitments. Digital modalities were not classified as independent prehabilitation modalities in this review, owing to limited use and overlap with other interventions, most commonly exercise [45]. Nonetheless, digital delivery represents a promising means of improving accessibility and reducing costs. Patients with mobility or transportation barriers may particularly benefit from tele-prehabilitation, which could extend care directly to patients’ homes. Evaluation of digital and hybrid models is warranted to assess feasibility, accessibility, and clinical effectiveness.

### Social Determinants of Health

Social determinants of health are another underexplored factor in prehabilitation implementation. Employment status, income, health literacy, and insurance coverage may all serve as barriers to participation, particularly when programs are costly, lengthy, or rigidly scheduled. Future investigations should examine how these determinants influence access, adherence, and outcomes, and design strategies to mitigate disparities.

### Publication Bias

Adoption of prehabilitation into routine surgical care is also influenced by the integrity and balance of the scientific literature. Publication bias is well recognized in clinical research, and although the extent of this bias in prehabilitation studies has not been quantified, the predominance of positive findings in the published literature is notable. Future investigations should assess the degree of publication bias to ensure balanced evidence synthesis.

### Clinical Integration

Elective surgery provides a unique opportunity for addressing uncontrolled or poorly managed chronic conditions through multidisciplinary collaboration and preoperative care coordination. Individualized medical optimization, including prehabilitation, has the potential to reduce preventable postoperative complications and should be considered for integration into standard preoperative planning. While the cost-effectiveness of such programs remains incompletely defined, efforts to implement affordable, accessible, and evidence-based prehabilitation protocols may serve to reduce disparities in surgical outcomes.

## Limitations and Future Directions

Several limitations of our review should be acknowledged. Categorization of prehabilitation modalities was based on published studies; however, interventions such as substance cessation and medical optimization may be more accurately considered elements of routine preoperative care rather than distinct prehabilitation modalities. In this regard, pain prehabilitation is limited in our medical literature, and presently, there is a paucity of studies. Similarly, digital prehabilitation may be best classified as a method of delivery rather than a standalone modality. Without this reclassification, overlaps arise, such as between digital and psychological interventions (e.g., virtual therapy) or digital and exercise interventions (e.g., wearable activity trackers). Future reviews should explicitly address modality overlaps and classify digital platforms as delivery mechanisms rather than modalities [46–48].

Another limitation was the predominance of cancer patients in the literature, many of whom underwent neoadjuvant chemotherapy prior to prehabilitation. This sequencing complicates interpretation, as improvements in postoperative outcomes may reflect the effects of neoadjuvant therapy rather than prehabilitation itself. Furthermore, cancer-specific outcomes were not frequently reported and, therefore, excluded from the analysis [49, 50]. Future research is needed to clarify the role of prehabilitation specifically in oncology populations.

Finally, as with all narrative reviews, the possibility of missed studies cannot be entirely excluded. Although multiple search strategies and keyword combinations were employed, some earlier studies may not have been captured if they were not indexed using the term “prehabilitation.” Given the use of broad search terms, the likelihood of missing recent publications is low; nevertheless, this limitation highlights the importance of ongoing literature updates as the field continues to evolve.

## Conclusion

Pain prehabilitation represents a promising and evolving area within perioperative medicine, with existing evidence suggesting associations with improved surgical outcomes. This narrative review identifies underutilization of prehabilitation strategies across several surgical specialties, highlighting important opportunities for future investigation. In addition, most studies to date have focused on single- or dual-modality interventions, underscoring the need for rigorous evaluation of comprehensive, multidisciplinary prehabilitation programs. Future research should prioritize both short- and long-term outcomes, including patient-centered measures such as quality of life, pain, and mental health.

## Supplementary Information

Below is the link to the electronic supplementary material.

Supplementary file1 Comprehensive metadata and manuscript-level details for 153 studies included after application of exclusion criteria. Data are organized by year of publication, surgical specialty, type of prehabilitation modality, study design, reported outcomes, and study conclusions. (XLSX 324 KB)

## Data Availability

All data necessary to reproduce the findings, including manuscript identifiers and analytical details, are provided in the supplementary table.

## References

[1] Wright S, Wiechula R, McLiesh P. The effectiveness of prehabilitation for adults having elective surgery: a systematic review protocol. JBI Database Syst Rev Implement Rep. 2016;14(2):78–92. 10.11124/jbisrir-2016-2460 27536795

[2] Weiser TG, Regenbogen SE, Thompson KD, et al. An estimation of the global volume of surgery: a modelling strategy based on available data. Lancet. 2008;372(9633):139–44. 18582931 10.1016/S0140-6736(08)60878-8

[3] Steiner CA, Karaca Z, Moore BJ, Imshaug MC, Pickens G. Surgeries in hospital-based ambulatory surgery and hospital inpatient settings, 2014. In: Healthcare cost and utilization project (HCUP) statistical briefs. Rockville (MD): agency for healthcare research and quality (US); 2017. 28722845

[4] Lawrence VA, Hazuda HP, Cornell JE, et al. Functional independence after major abdominal surgery in the elderly. J Am Coll Surg. 2004;199(5):762–72. 15501119 10.1016/j.jamcollsurg.2004.05.280

[5] Hoff SR, Schroeder JW Jr, Rastatter JC, Holinger LD. Supraglottoplasty outcomes in relation to age and comorbid conditions. Int J Pediatr Otorhinolaryngol. 2010;74(3):245–9. 20022388 10.1016/j.ijporl.2009.11.012

[6] Wu C, Evans I, Joseph R, et al. Comorbid conditions in kidney transplantation: association with graft and patient survival. J Am Soc Nephrol. 2005;16(11):3437–44. 16176999 10.1681/ASN.2005040439

[7] Loewenstern J, Kessler RA, Caridi J. Diabetes comorbidity increases risk of postoperative complications in traumatic thoracic vertebral fracture repair: a propensity score matched analysis. World Neurosurg. 2019;121:e792-7. 30312819 10.1016/j.wneu.2018.09.225

[8] Wynter-Blyth V, Moorthy K. Prehabilitation: preparing patients for surgery. BMJ. 2017;358:j3702 (**Published 2017 Aug 8**). 28790033 10.1136/bmj.j3702

[9] Santa Mina D, Scheede-Bergdahl C, Gillis C, Carli F. Optimization of surgical outcomes with prehabilitation. Appl Physiol Nutr Metab. 2015;40(9):966–9 (****(summarizes role of prehabilitation in surgical outcomes)**). 26300015 10.1139/apnm-2015-0084

[10] Bordes P, Laboute E, Bertolotti A, et al. No beneficial effect of bracing after anterior cruciate ligament reconstruction in a cohort of 969 athletes followed in rehabilitation. Ann Phys Rehabil Med. 2017;60(4):230–6. 28259710 10.1016/j.rehab.2017.02.001

[11] Dannenmaier J, Ritter S, Jankowiak S, Kaluscha R, Krischak G. Erwerbsstatus nach einer bandscheibenoperation: was bewirkt eine anschlussrehabilitation? [Return to Work after Disk Surgery - Influenced by Rehabilitation?]. Rehabilitation (Stuttg). 2018;57(1):38–47. 28746952 10.1055/s-0043-107928

[12] Marcotte JH, Patel K, Desai R, et al. Acute kidney injury following implementation of an enhanced recovery after surgery (ERAS) protocol in colorectal surgery. Int J Colorectal Dis. 2018;33(9):1259–67. 29808304 10.1007/s00384-018-3084-9

[13] Fuglsang S, Heiberg J, Hjortdal VE, Laustsen S. Exercise-based cardiac rehabilitation in surgically treated type-A aortic dissection patients. Scand Cardiovasc J. 2017;51(2):99–105. 27808563 10.1080/14017431.2016.1257149

[14] Della Villa S, Kon E, Filardo G, Ricci M, Vincentelli F, Delcogliano M, et al. Does intensive rehabilitation permit early return to sport without compromising the clinical outcome after arthroscopic autologous chondrocyte implantation in highly competitive athletes? Am J Sports Med. 2010;38:68–77. 20051508 10.1177/0363546509348490

[15] Feroci F, Kröning KC, Lenzi E, Moraldi L, Borrelli A, Scatizzi M. Lo sviluppo di un protocollo di fast track surgery dopo colectomia laparoscopica in una unità di chirurgia generale [The development of a fast track surgery program after laparoscopic colonic surgery in a General Surgery Unit]. Minerva Chir. 2010;65(2):127–36. 20548268

[16] Prehabilitation R. Revocation in the army. Br Med J. 1946;1:192–7. 20989832

[17] Banugo P, Amoako D. Prehabilitation. BJA Educ. 2017;17:401–5.

[18] Tew GA, Ayyash R, Durrand J, Danjoux GR. Clinical guideline and recommendations on preoperative exercise training in patients awaiting major non-cardiac surgery. Anaesthesia. 2018;74:750–68. 10.1111/anae.14177 29330843

[19] Le Roy B, Selvy M, Slim K. The concept of prehabilitation: what the surgeon needs to know. J Visc Surg. 2016;153:109–12. 26851994 10.1016/j.jviscsurg.2016.01.001

[20] Mayo NE, Feldman L, Scott S, et al. Impact of preoperative change in physical function on postoperative recovery: argument supporting prehabilitation for colorectal surgery. Surgery. 2011;150:505–14. 21878237 10.1016/j.surg.2011.07.045

[21] Kassin MT, Owen RM, Perez SD, et al. Risk factors for 30-day hospital readmission among general surgery patients. J Am Coll Surg. 2012;215:322–30. 22726893 10.1016/j.jamcollsurg.2012.05.024 PMC3423490

[22] Scheede-Bergdahl C, Minnella EM, Carli F. Multimodal prehabilitation: addressing the why, when, what, who and where next. Anaesthesia. 2019;74:20–6. 30604416 10.1111/anae.14505

[23] Gillis C, Fenton TR, Sajobi TT, et al. Trimodal prehabilitation for colorectal surgery attenuates post-surgical losses in lean body mass: a pooled analysis of randomized controlled trials. Clin Nutr. 2019;38:1053–60. 30025745 10.1016/j.clnu.2018.06.982

[24] Bruns ERJ, van den Heuvel B, Buskens CJ, et al. The effects of physical prehabilitation in elderly patients undergoing colorectal surgery: a systematic review. Colorectal Dis. 2016;18:O267–77. 27332897 10.1111/codi.13429

[25] Knight JB, Subramanian H, Sultan I, Kaczorowski DJ, Subramaniam K. Prehabilitation of cardiac surgical patients, part 1: anemia, diabetes mellitus, obesity, sleep apnea, and cardiac rehabilitation. Semin Cardiothorac Vasc Anesth. 2022;26(4):282–94. 36006868 10.1177/10892532221121118

[26] Rouleau CR, Chirico D, Hauer T, Kidd W, Arena R, Aggarwal SG. An observational study examining utilization of prehabilitation and its association with postoperative cardiac rehabilitation participation and risk factors following coronary artery bypass grafting. Int J Cardiol. 2022;362:28–34. 35526657 10.1016/j.ijcard.2022.05.006

[27] Santa Mina D, Clarke H, Ritvo P, et al. Effect of total-body prehabilitation on postoperative outcomes: a systematic review and meta-analysis. Physiotherapy. 2014;100:196–207. 24439570 10.1016/j.physio.2013.08.008

[28] Ebner F, Schulz SVW, de Gregorio A, et al. Prehabilitation in gynecological surgery? What do gynecologists know and need to know. Arch Gynecol Obstet. 2018;297:27–31. 29075851 10.1007/s00404-017-4565-8

[29] Centemero A, Rigatti L, Giraudo D, et al. Preoperative pelvic floor muscle exercise for early continence after radical prostatectomy: a randomized controlled study. Eur Urol. 2010;57:1039–43. 20227168 10.1016/j.eururo.2010.02.028

[30] Moran J, Guinan E, McCormick P, et al. The ability of prehabilitation to influence postoperative outcome after intra-abdominal operation: a systematic review and meta-analysis. Surgery. 2016;160:1189–201. 27397681 10.1016/j.surg.2016.05.014

[31] Li C, Carli F, Lee L, et al. Impact of a trimodal prehabilitation program on functional recovery after colorectal cancer surgery: a pilot study. Surg Endosc. 2013;27:1072–82. 23052535 10.1007/s00464-012-2560-5

[32] Valkenet K, van de Port IG, Dronkers JJ, de Vries WR, Lindeman E, Backx FJ. The effects of preoperative exercise therapy on postoperative outcome: a systematic review. Clin Rehabil. 2011;25:99–111. 21059667 10.1177/0269215510380830

[33] Halloway S, Buchholz SW, Wilbur J, Schoeny ME. Prehabilitation interventions for older adults: an integrative review. West J Nurs Res. 2015;37:103–23. 25255975 10.1177/0193945914551006

[34] Ridge CA, McErlean AM, Ginsberg MS. Epidemiology of lung cancer. Semin Intervent Radiol. 2013;30:93–8. 24436524 10.1055/s-0033-1342949 PMC3709917

[35] Cabilan CJ, Hines S, Munday J. The impact of prehabilitation on postoperative functional status, healthcare utilization, pain, and quality of life: a systematic review. Orthop Nurs. 2016;35:224–37. 27441877 10.1097/NOR.0000000000000264

[36] Baimas-George M, Watson M, Elhage S, Parala-Metz A, Vrochides D, Davis BR. Prehabilitation in frail surgical patients: a systematic review. World J Surg. 2020;44:3668–78 (*****(important systematic review on prehabilitation in frail surgical patients)**). 32656590 10.1007/s00268-020-05658-0

[37] Minnella EM, Coca-Martinez M, Carli F. Prehabilitation: the anesthesiologist’s role and what is the evidence? Curr Opin Anaesthesiol. 2020;33:411–6. 32371632 10.1097/ACO.0000000000000854

[38] Asoh T, Tsuji H. Preoperative physical training for cardiac patients requiring non-cardiac surgery. Jpn J Surg. 1981;11:251–5. 7289232 10.1007/BF02468764

[39] Durrand J, Singh SJ, Danjoux G. Prehabilitation. Clin Med. 2019;19:458–64. 10.7861/clinmed.2019-0257 PMC6899232 31732585

[40] Makary MA, Segev DL, Pronovost PJ, et al. Frailty as a predictor of surgical outcomes in older patients. J Am Coll Surg. 2010;210:901–8. 20510798 10.1016/j.jamcollsurg.2010.01.028

[41] Gillis C, Li C, Lee L, et al. Prehabilitation versus rehabilitation: a randomized control trial in patients undergoing colorectal resection for cancer. Anesthesiology. 2014;121:937–47. 25076007 10.1097/ALN.0000000000000393

[42] Santa Mina D, Hilton WJ, Matthew AG, et al. Prehabilitation for radical prostatectomy: a multicentre randomized controlled trial. Surg Oncol. 2018;27:289–98. 29937184 10.1016/j.suronc.2018.05.010

[43] Milios JE, Ackland TR, Green DJ. Pelvic floor muscle training in radical prostatectomy: a randomized controlled trial of the impacts on pelvic floor muscle function and urinary incontinence. BMC Urol. 2019;19:116. 31729959 10.1186/s12894-019-0546-5 PMC6858748

[44] Hrubá S, Chovanec M, Čada Z, et al. The evaluation of vestibular compensation by vestibular rehabilitation and prehabilitation in short-term postsurgical period in patients following surgical treatment of vestibular schwannoma. Eur Arch Otorhinolaryngol. 2019;276:2681–9. 31187238 10.1007/s00405-019-05503-8

[45] Magnusson M, Karlberg M, Tjernström F. “PREHAB”: vestibular prehabilitation to ameliorate the effect of a sudden vestibular loss. NeuroRehabilitation. 2011;29:153–6. 22027076 10.3233/NRE-2011-0689

[46] Baldini G, Ferreira V, Carli F. Preoperative preparations for enhanced recovery after surgery programs: a role for prehabilitation. Surg Clin North Am. 2018;98:1149–69. 30390849 10.1016/j.suc.2018.07.004

[47] Mouch CA, Kenney BC, Lorch S, et al. Statewide prehabilitation program and episode payment in medicare beneficiaries. J Am Coll Surg. 2020;230:306-313.e6. 31812662 10.1016/j.jamcollsurg.2019.10.014

[48] Dholakia J, Cohn DE, Montemorano L, Straughn JM, Dilley SE. Prehabilitation is a cost-saving method with improved outcomes for medically frail patients undergoing surgery for epithelial ovarian cancer: a cost-effectiveness analysis. Gynecol Oncol. 2020;159:74. 10.3802/jgo.2021.32.e92 PMC8550928 34708594

[49] Tsai MH, Porter JC, Adams DC. The denominator in value-based health care: Porter’s hidden costs. Anesth Analg. 2018;127:317. 29734241 10.1213/ANE.0000000000003401

[50] Barberan-Garcia A, Ubre M, Pascual-Argente N, et al. Post-discharge impact and cost-consequence analysis of prehabilitation in high-risk patients undergoing major abdominal surgery: secondary results from a randomised controlled trial. Br J Anaesth. 2019;123:450–6. 31248644 10.1016/j.bja.2019.05.032

